# How to better balance academic achievement and learning anxiety from time on homework? A multilevel and classification and regression tree analyses

**DOI:** 10.3389/fpsyg.2023.1130274

**Published:** 2023-05-09

**Authors:** Xiaopeng Wu, Rongxiu Wu, Carol Hanley, Hongyun Liu, Jian Liu

**Affiliations:** ^1^Faculty of Education, Northeast Normal University, Changchun, China; ^2^Department of Science Education, Harvard-Smithsonian Center for Astrophysics, Harvard University, Cambridge, MA, United States; ^3^College of Agriculture, Food and Environment, University of Kentucky, Lexington, KY, United States; ^4^Beijing Key Laboratory of Applied Experimental Psychology, Faculty of Psychology, Beijing Normal University, Beijing, China; ^5^Collaborative Innovative Center of Assessment Toward Basic Education Quality, Beijing, China

**Keywords:** time on homework, academic achievement, learning anxiety, multilevel analysis, classification and regression tree

## Abstract

Using education survey data from 153, 317 Grade 4 students and 150, 040 Grade 8 students in China, this study examined the relationship between time on homework and academic achievement and learning anxiety with hierarchical linear modeling (HLM) and classification and regression tree (CART) approaches. With a classification of time spent on homework into four related variables, this study found that, firstly, time spent on in-school homework during weekdays had positive effects on students’ achievement for both grades, and the positive effect was stronger for Grade 8 students than Grade 4 students. Moreover, a maximum of 1 h was recommended for Grade 4 students. Secondly, time spent on out-of-school homework on weekdays was negatively correlated with students’ academic achievement and positively with learning anxieties. It had greater detrimental effect on Grade 8 than Grade 4. Thirdly, Grade 8 students were encouraged to have more out-of-school homework on weekend with more than 2.8 h on average recommended. It was expected to complement extant studies and provide the practical findings for teachers, practitioners and school policy makers in making any homework assignment planning or conducting interventions.

## 1. Introduction

Extensive literature confirms the effectiveness of homework in students’ academic achievement and engagement (Cooper, 1989; Keith et al., 2004; Cooper et al., 2006; Katz et al., 2012; Fan et al., 2017). In the 2022 report from the National Assessment of Educational Progress (NAEP), it reports that time spent on homework was increasing for different age groups (9-year-old, 13-year-old, and 17-year-old) from 1984 through 2012 (U.S. Department of Education, 2022). Due to its complicated characteristics (e.g., frequency, purpose, and amount), homework has been viewed as one trigger for excessive pressure and anxiety that impedes students from success (Cooper, 2015; Hong et al., 2015). With the facilitating effect of homework, whether there is a tradeoff between students’ academic achievement and learning anxiety has become an urgent issue for researchers to take a closer examination (Cooper, 1989; Cooper and Valentine, 2001).

However, in previous research, though large body of homework related meta-analyses exist (e.g., Cooper et al., 2006; Fan et al., 2017), homework has been widely considered as a variable linearly related with the outcome (e.g., academic achievement) with support from schools and families. To say it alternatively, they stated that the more (the less) homework is, the better the student outcome. In addition, studies do exist that discuss the possible curvilinear relationship between homework and outcomes (Cooper and Valentine, 2001). Nonetheless, there are rarely any studies that have taken homework and its two closely related outcomes, which are academic achievement and learning anxiety together into one analysis. Considering its significance and less investigation of the topic, this study was aimed to explore deeper the relationship between students’ time on homework and their academic achievement and learning anxiety.

## 2. Theoretical background

### 2.1. Homework and academic achievement

Homework, “tasks assigned to students by teachers to be carried out during the non-school hours” (Cooper, 1989, p. 7), is an essential instructional strategy that aims to supplement students’ learning in school. A large body of existing studies have investigated the relationship between time spent on homework and academic achievement (Cooper et al., 2001; Trautwein et al., 2002; Trautwein and Köller, 2003; Cooper et al., 2006; Trautwein, 2007; Dettmers et al., 2009; Fan et al., 2017; Flunger et al., 2021). Most researchers have considered homework-achievement relationship as linear, and the conclusions varied greatly. Cooper et al. (2006) synthesized US studies from 1987 to 2003 across subjects and found the positive relationship between homework and achievement was stronger for Grades 7–12 than that for K-6. Fan et al. (2017) indicated a small positive relationship between homework time and math and science from 1986 to 2015 across the US, Europe, and Asia. Negative correlations were also identified throughout the studies. For instance, using nationally representative *Program for International Student Assessment* (PISA) German and American samples, Trautwein (2007) indicated negative relationships between homework time and math achievement. Studies showed no correlations were also found, for instance, using a sample of 1,976 middle school students, Trautwein et al. (2002) indicated that the amount of homework and time spent on homework were not correlated with students’ achievement.

There are also researchers who support a non-linear relationship between homework time and achievement. They believe that time on homework can reach a plateau at which increases in time has only a marginal effect on learning outcome (Fredrick and Walberg, 1980; Keith, 1982; Cooper and Valentine, 2001). Keith (1982) stated that the effects of time spent on homework may be curvilinear, with each additional hour showing a smaller payoff in achievement. Cooper and Valentine (2001) reported that for junior high school students, minimal amount of time on homework can have positive relationship with achievement but the effect disappeared entirely after students reported doing homework between 1 and 2 h each night. For high school students, suggested a time range between 1.5 and 2.5 h per night as optimal. One theoretical foundation can support these findings is the *Opportunity to Learn* paradigm (Carroll, 1963; Paschal et al., 1984). It recognizes that learning does yield positive learning outcomes but can only up to the amount needed to learn new materials. The effect of learning occurs at the conjunction of *time spent on learning* and *time needed to learn*.

### 2.2. Homework and learning anxiety

Except for the facilitating effect of homework on students’ academic achievement, contemporary learning theories also expressed the concern of the negative impact such as anxiety that homework may bring to students, which can greatly associate with homework effectiveness and undermine the positive effects of homework (Cheung and Leung-Ngai, 1992; Conner et al., 2009; Katz et al., 2012; Galloway et al., 2013; Hong et al., 2015). Galloway et al. (2013) found students in upper middle-class on average spent 3 h of homework per night, but beyond 3 h, students experienced more academic stress and physical health problems. Michaud et al. (2015) also found more hours spent on homework was associated with physical health problems and they have emphasized an extra attention should be paid to stressful mental work due to homework duration. To better measure stress experienced by doing homework, Katz et al. (2012) validated a relevant homework stress scale, in which a series of psychological reactions such as tension, distraction and irritability caused by doing homework were depicted. Hong et al. (2015) described anxiety toward homework as one kind of cognitive concerns about the negative expectations, and potential consequences of homework performance. Children with different backgrounds may exhibit different intensities of anxieties in doing homework as well. Xiao et al. (2022) identified that time spent on homework were associated with higher scores in anxiety for dyslexic children compared to normal children. In our proposed study, we used the scale learning anxiety to refer to the general anxiety that students experience in schools. It mainly refers to the situation-specific anxiety responding to the learning-related situations (e.g., general academic, test, or social performance) in school context (Wu et al., 2023).

### 2.3. Classifications of homework

When and where homework is completed have been identified as factors impacting students’ academic achievement (Keith et al., 2004). Some teachers have argued that there existed different effects of homework completion in schools or out of schools (Cooper and Valentine, 2001; Keith et al., 2004). In Cooper and Valentine (2001)’s meta-analysis, they showed some slight effect for out-of-school homework over in-school supervised homework. Keith et al. (2004) discovered that time spent doing homework in school had no effect while time spent doing homework out of school had quite a substantial effect on high school students’ grades. So far, very limited quantitative studies have taken the homework characteristics into consideration while conducting relevant research. What’s more, in current learning environment, students have been assigned varieties of formats of out-of-school homework except for those assigned by schoolteachers, and they even need to squeeze their weekend time working on the in-school and out-of-school homework as well (Mau and Lynn, 2000; Hagger et al., 2015). To have a deeper understanding of the effect of homework on students’ academic achievement and learning anxiety and provide more practical findings for schoolteachers, parents, and policymakers, this study made a more nuanced classification of time spent on homework and compared their differences of effects on academic achievement and learning anxiety. Specifically in our study, homework time has been classified into (1) time on in-school homework on weekdays; (2) time on in-school homework on weekends; (3) time on out-of-school homework on weekdays; and (4) time on out-of-school homework on weekends.

### 2.4. The present study

Informed by theoretical framework and related previous studies, below were two research hypotheses that we proposed for the current study. First, it was hypothesized that school-level homework related variables relate with students’ academic achievement and learning anxieties. Second, it was hypothesized that thresholds and range of time existed for optimal academic achievement and minimal learning anxiety. Since students in different grades may exhibit various characteristics (Cooper, 1989; Cooper et al., 1998; Cooper and Valentine, 2001; Cooper et al., 2006; Fan et al., 2017), a sample of students from both Grade 4 and Grade 8 in a national large-scale dataset were applied in this study. Using statistical technique *Hierarchical Linear Modeling* (HLM), we first examined how these homework related variables impact students’ academic achievement and learning anxieties within the school context (De Jong et al., 2000; Trautwein, 2007; Trautwein et al., 2009). To closely capture local variable manifestations and experiment with the data to derive insights (Ma, 2005), we then followed up with *classification and regression tree* (CART) to investigate whether there existed any thresholds or ranges of time for students to spend on homework related variables to reach an optimal achievement outcome while minimizing students’ learning anxiety. Specifically, the following research questions were to be answered:

1. Were schools responsible for the variation in students’ academic achievement and learning anxiety for both Grade 4 and 8? If there were school effects, what specific school-level homework related variable(s) showed statistically significant relationships with the outcome variables?
2. What were the local manifestations of thresholds or range of time for students to spend on homework variables related with optimal achievement outcome and minimal students’ learning anxiety?

## 3. Method

### 3.1. Data source and procedure

With high prevalence and long tradition in homework, China has been a “frontier” offering plenty of opportunities to examine the effect of homework (Hong et al., 2009). This study used data from the *Program of Regional Education Assessment* implemented annually in China by the *Collaborative Innovation Center of Assessment toward Basic Education Quality* (CICA-BEQ) at one university in Beijing. Through conducting standardized achievement tests toward students, teachers, and principals, the main purpose of the program was to understand the changes in teaching quality and students’ learning outcomes in different locations of China (Liu et al., 2021). Variables and scales used in this national survey have all gone through rigorous reliability and validity examination. All participators were informed about goals of the study and assured that their data would be used for scientific purposes only. In accordance with the recommendations of the university, the administration was implemented with the written informed consent from the participants. *Institutional Review Board* (IRB) was also approved by the university.

The current dataset consisted of 158,318 Grade 4 students (Male: 54.10%, Female: 45.90%) from 1,739 elementary schools and 158,277 Grade 8 students (boys: 53.30%, girls: 46.70%) from 1,065 secondary schools in various provinces of China in 2016. More demographic information can be seen in Table 1.

**Table 1:** Demographic information of Grade 4 and Grade 8.

Variables		Grade 4 (*N* = 158,318)		Grade 8 (*N* = 158,277)	
		*N*	%	*N*	%
Gender	Male	85,626	53.5	84,434	53.30
	Female	72,692	45.4	73,843	46.7
		*M*	*SD*	*M*	*SD*
*ISES* (standardized)		−0.02	0.75	0.00	0.75
Academic scores		552.13	85.76	538.23	81.95
Learning anxiety		4.10	2.75	4.79	2.74
*ISHWD*		1.59	1.23	2.38	1.14
*ISHWE*		2.03	1.30	2.96	1.27
*OSHWD*		0.74	1.06	0.55	0.92
*OSHWE*		2.22	2.63	0.78	1.05

*ISHWD* is in-school homework time on weekdays, *ISHWE* is in-school homework time on weekends, *OSHWD* is out-of-school homework time on weekdays, and *OSHWE* is out-of-school homework time on weekends.

### 3.2. Variables

Dependent variables used in this study were two separate variables *students’ academic achievement* and *learning anxiety*. Independent variables were classified into student-level and school-level characteristics. Student-level variables included four homework related variables, which were *in-school homework time on weekdays, in-school homework time on weekends, out-of-school homework time on weekdays, out-of-school homework time on weekends*, as well as two control variables *gender* and *individual socioeconomic status (ISES)*. School-level variables included four relevant school-level homework variables; school context variables, which were *school size* and *mean SES*; and school climate variables *teacher-student relationship, peer relationship, school belongingness* and *schoolteacher leadership*. The variables were specifically described as below.

#### 3.2.1. Students’ academic achievement

Since students’ academic achievement is relatively stable across all subjects (Chang et al., 2014), in this study, the students’ average scores across subjects were used as students’ academic achievement. For Grade 4, students’ academic score was an average of main subjects’ scores reading, science, and math (*M* = 552.13, *SD* = 85.76); for Grade 8, students’ academic score was an average of students’ reading, science, math, and English scores (*M* = 538.23, *SD* = 81.95).

#### 3.2.2. Learning anxiety

Anxiety literature showed that working on homework for a long time easily result in poor homework performance and learning anxiety. Designed by CICA-BEQ (Liu et al., 2021), learning anxiety was measured with eight dichotomous items for both grades, which were “Will you feel nervous when teachers raise questions in class?,” “Will you feel nervous when knowing an ‘exam’ is around the corner?,” “Will you feel nervous if you do not do well in your exam?,” “Will you feel nervous if you do not do well in your daily learning?,” “Will you feel nervous about the scores after taking the exam?,” “Will you feel nervous that you could not do well before taking the exam?,” “Will you feel nervous that you cannot complete the tasks before the work starts?” and “Will you feel nervous to present in class?” (Cronbach’s alpha *a* = 0.86).

#### 3.2.3. Student-level characteristics

Variables related to students’ time spent on homework included four specific student-level variables, which were (1) *in-school homework time on weekdays (ISHWD)*: what is the average amount of time you spend on in-school homework on weekdays; (2) *in-school homework time on weekends (ISHWE)*: what is the average amount of time you spend on in-school homework on weekends; (3) *out-of-school homework time on weekdays (OSHWD)*: what is the average amount of time you spend on out-of-school homework on weekdays; and (4) *out-of-school homework time on weekends (OSHWE)*: what is the average amount of time you spend on out-of-school homework on weekends. There were five response options for each of the variable: (1) never, (2) under 3 h (3 h is not included), (3) 3 to 6 h (6 h is not included), (4) 6 to 8 h (8 h is not included), and (5) 8 h and more. To have a more accurate analysis of the variable in the model, these responses have been sequentially recoded into (1) 0 h, (2) 1.5 h, (3) 4.5 h, (4) 7.5 h, and (5) 10 h. In addition, *Gender* (“Are you female,” Yes = 1, No = 0) and *ISES* (derived from three relevant variables, Liu et al., 2021) were also included as control variables in the student level.

#### 3.2.4. School-level characteristics

School characteristics were classified into school context variables, including *school size* (true school level values) and *mean SES* (average mean from the student level SES). School climate variables were *teacher-student relationship (TSR)* (Cronbach’s alpha *a* = 0.93) measured with five items (e.g., *I get along with the teachers*); *peer relationship (PEER)* (Cronbach’s alpha *a* = 0.85) measured with 10 items (e.g., *I am happy with my schoolmates*); *school belongingness (SBEL)* (Cronbach’s alpha *a* = 0.85) measured with four items (e.g., *I like being in the school*); *schoolteacher leadership (TLEA)* (Cronbach’s alpha *a* = 0.85) measured with 14 items (e.g., *I am fair to every teacher regarding the issue of teachers’ assessment*). These school climate variables were all measured using 5-point Likert scale “strongly disagree,” “disagree,” “not sure,” “agree,” and “strongly agree.” The four relevant school-level homework variables *SISHWD, SISHWE, SOSHWD*, and *SOSHWE* were the averages of student-level homework related variables.

The correlations among student-level variables were examined to assess multicollinearity. The ranges of correlations were between 0.01 and 0.66, therefore, no correlations were large enough to warrant caution in either Grade 4 or 8 analyses (Table 2). In addition, for the convenience of data analysis and comparison, continuous variables at the student and school levels were all standardized with mean of 0 and standard deviation of 1 in HLM and original means of independent variables were used in CART analysis.

**Table 2:** Correlations for all student-level variables in Grades 4 and 8.

	1	2	3	4	5	6	7	8
1. Average score	1	−0.04**	0.32**	−0.18**	−0.11**	0.04**	−0.09**	−0.05**
2. Gender	−0.15**	1	−0.02**	−0.02**	0.01**	0.01**	0.01**	0.02
3. *ISES*	0.25**	0.01**	1	−0.16**	−0.05**	0.01	0.09**	0.09**
4. Learning anxiety	−0.08**	−0.10**	−0.09**	1	0.13**	0.10**	0.05**	0.01**
5. *ISHWD*	0.14**	−0.05**	0.10**	0.10**	1	0.55**	0.22**	0.11**
6. *ISHWD*	0.28**	−0.09**	0.19**	0.09**	0.56**	1	0.15**	0.10**
7. *OSHWD*	−0.05**	0.03**	0.10**	0.04**	0.20**	0.12**	1	0.40**
8. *OSHWE*	0.08**	−0.02**	0.17**	0.04**	0.20**	0.21**	0.66**	1

**p < 0.01, ***p < 0.001. *ISHWD* is in-school homework time on weekdays, *ISHWE* is in-school homework time on weekends, *OSHWD* is out-of-school homework time on weekdays, and *OSHWE* is out-of-school homework time on weekends. The left below diagonal shows the correlations in Grade 8, and the right upper diagonal shows the correlations in Grade 4.

### 3.3. Analytical approach

#### 3.3.1. Hierarchical linear analysis

As an advanced statistic, HLM can take the data analyses into the multilevel context and partition the total variance of outcome variable into within- and between-school variances. It was employed first to develop the multilevel models that estimated students’ outcome variables *academic achievement* and *learning anxiety* one by one for each grade. Null models were conducted first in which no explanatory variables at either the student- or school-level were included and then further expanded to include predictors at both levels. The *intraclass correlation coefficient* (ICC), an indicator of whether there is evidence of clustering or nesting in a model, can be obtained from the null model (Raudenbush and Bryk, 2002). All variables were grand mean-centered except for *gender*, and the coefficients from the student-level model were fixed. Pairwise deletion was conducted for all student-level and listwise deletion for the school-level analyses was conducted to deal with missing data. Sampling weights were also added to ensure each sample make an equal contribution to parameter estimation. Statistical software SPSS (IBM Corp, 2017) was used for data management and HLM 8.0 (Raudenbush et al., 2019) was used for hierarchical model construction and analysis.

*Null model:*

Level 1*: AcademicScore_ij_* = *β_0j_* + *r_ij_.*

& *LearningAnxiety_ij_* = *β_0j_* + *r_ij_.*

Level 2: *β_0j_* = *γ_00_* + *u_0j_.*

HLM then estimated the student and school effects with adjustments for the control variables in the full model. The level-1 model was the student-level model, with a set of individual linear regressions, one for each student. Students’ average academic score and learning anxiety were separately regressed on *gender, ISES, ISHWD, ISHWE, OSHWD,* and *OSHWE.* The intercept of each regression represented the average measure of students’ academic scores or learning anxieties within each school, adjusted for student characteristics in that school. The slope of the regression represented the relationship between the outcome variables with each specific variable in that model. These parameters were then used as outcome measures in the second-level model. The second level represented the school-level model, a set of linear regressions that regressed the average school measures of students’ academic scores and learning anxieties on the four school-level predicted variables *SISHWD, SISHWE, SOSHWD* and *SOSHWE* along with school context and climate variables. Assumptions for the HLM models were also examined. For instance, (a) residuals at the student and school levels have a normal distribution with a population mean of zero and constant variance, and (b) residuals at the school level are independent across the schools. No serious violation of HLM assumptions were detected.

*Full model:*


$$\begin{array}{l} \mathrm{Level}\;1:\\ \begin{array}{cc} \begin{array}{l} AcademicScore\\ \;\left(LearningAnxiety_{ij}\right) \end{array} & \begin{array}{l} =\beta_{0j}+\beta_{1j}\;\left(ISES_{ij}\right)+\beta_{2j}\;\left(GENDER_{ij}\right)\\ +\beta_{3j}\;\left({\mathrm{ISHWD}}_{ij}\right)+\beta_{4j}\;\left({\mathrm{ISHWE}}_{ij}\right)\\ +\beta_{5j}\;\left({\mathrm{OSHWD}}_{ij}\right)+\beta_{6j}\;\left({\mathrm{OSHWE}}_{ij}\right)+r_{ij} \end{array} \end{array} \end{array}$$

$$\begin{array}{l} \mathrm{Level}\;2:\beta_{0j}=\gamma_{00}+\gamma_{01}\;\left(SCHSIZE_{j}\right)+\gamma_{02}\;\left(SCHSES_{j}\right)\\ +\gamma_{03}\;\left(TSR_{j}\right)+\gamma_{04}\;\left(PEER_{j}\right)+\gamma_{05}\;\left(TLEA_{j}\right)\\ +\gamma_{06}\;\left(SBEL_{j}\right)+\gamma_{07}\;\left({\mathrm{SISHWD}}_{j}\right)+\gamma_{08}\;\left({\mathrm{SISHWE}}_{j}\right)\\ +\gamma_{09}\;\left({\mathrm{SOSHWD}}_{j}\right)+\gamma_{010}\;\left({\mathrm{SOSHWE}}_{j}\right)+u_{0j}\;\\ \beta_{1j}=\gamma_{10}\;\beta_{2j}=\gamma_{20}\;\beta_{3j}=\gamma_{30}\;\beta_{4j}=\gamma_{40}\;\\ \beta_{5j}=\gamma_{50}\;\beta_{6j}=\gamma_{60} \end{array}$$

#### 3.3.2. Classification and regression tree analysis

The study followed up with CART analyses which can identify all nonlinear interactive relationships between variables with no need for pre-specification in the models (Breiman et al., 1984). It formulates mutually exclusive and exhaustive groups through the decomposition of interactions among independent variables, so cases are homogeneous within each group and heterogeneous between groups. Therefore, researchers can easily capture local variable manifestations of interest and experiment with the data to derive insights that eventually might be pieced together to formulate a theory (Ma, 2005). This process provides researchers with an instant picture of the interaction effects of key explanatory measures (Berk, 2016). CART works by performing binary splitting of groups based on impurity, which measures how persons in a node vary on an outcome measure. The splitting and pruning process starts with the most influential root node to the least, and each explanatory variable is examined for how well it splits the participants into two groups in the child nodes. The nodes that cannot be split further are called terminal nodes. Terminal nodes are always worth noting since they often show dramatically different outcomes depending on the outcome variable. To avoid a tree with too many branches, we decided to trim the CART with three to four levels in our study. Focusing the second research question in the study, the two dependent variables *academic score* and *learning anxiety* and the independent school-level homework related variables which showed statistically significance in HLM were selected to be used in the CART analysis.

## 4. Results

### 4.1. HLM analysis

The null models with only dependent variables included were tested one by one, and ICC values were obtained. Table 3 showed that substantial number of variances in students’ average *academic score* and *learning anxiety* were attributable to schools for both grades. Specifically, schools were attributable for 29% of the variance in students’ academic scores for Grade 4 and 35% for Grade 8; and 11% of the variance in students’ learning anxiety for Grade 4 and 7% for Grade 8. Therefore, all ICCs demonstrated that there was a necessity for the two-level modeling in explaining the outcome variables.

**Table 3:** Proportions of variance in students’ academic score and learning anxiety within and between schools.

Outcome variables	Grade level	Source of variation	ICCs
Students’ academic score	Grade 4	Within schools	0.71
		Between schools	0.29
	Grade 8	Within schools	0.65
		Between schools	0.35
Students’ learning anxiety	Grade 4	Within schools	0.89
		Between schools	0.11
	Grade 8	Within schools	0.93
		Between schools	0.07

ICC, Intraclass correlation coefficient.

The full models were then tested with all the independent variables and controlling variables included. Table 4 showed the student- and school-level effects of academic score and learning anxiety. Since homework was the work assigned and monitored by teachers and school administrations rather than the students themselves (Trautwein and Köller, 2003; Cooper et al., 2006), its effect on the school level was the focus in our study. Overall, for Grades 4 and 8, *SISHWD* showed significant positive relationships with students’ academic achievement for Grade 4 (*B* = 3.30, *SE* = 1.60) and Grade 8 (*B* = 5.40, *SE* = 2.23) after controlling for all other variables in the model and the effect for Grade 8 was even higher than that for Grade 4. In addition, *SISHWD* did not associate with students’ learning anxieties for Grades 4 or 8 either. However, for Grade 4, *SISHWE* was statistically positively correlated with students’ academic achievement (*B* = 3.62, *SE* = 1.50), but it greatly associated with students’ learning anxieties (*B* = 0.17, *SE* = 0.04). Moreover, in terms of *SOSHWD*, it was negatively correlated with students’ academic achievement for both grades: Grade 4 (*B* = −6.04, *SE* = 1.49) and Grade 8 (*B* = −14.63, *SE* = 2.13) though it did not relate with or positively relate with students’ learning anxieties for either of the grade. In terms of *SOSHWE*, it had a statistically positive effect on students’ academic achievement (*B* = 14.59, *SE* = 1.92) and did not relate with learning anxiety for Grade 8 students.

**Table 4:** Results of student and school effects on students’ academic achievement and learning anxiety.

Outcome variable	Academic achievement				Learning anxiety			
Effects of student characteristics	Grade 4		Grade 8		Grade 4		Grade 8	
	*B*	*SE*	*B*	*SE*	*B*	*SE*	*B*	*SE*
Gender	−4.10**	0.57	−20.74**	0.63	−0.13**	0.02	−0.52**	0.02
*ISES*	19.49**	0.49	6.37**	0.65	−0.54**	0.02	−0.36**	0.02
*ISHWD*	−9.49**	0.38	−1.33*	0.47	0.16**	0.01	0.17**	0.01
*ISHWE*	9.25**	0.36	15.37**	0.48	0.11**	0.01	0.09**	0.02
*OSHWD*	−6.62**	0.50	−10.80**	0.41	0.07**	0.01	0.05**	0.01
*OSHWE*	2.70*	1.09	5.64**	0.44	−0.03*	0.01	0.00	0.01
Effects of school characteristics								
School SES	10.25**	1.25	33.95**	4.43	0.13**	0.03	−0.07	0.07
Peer relationship	14.15**	1.64	4.90*	2.01	−0.25**	0.04	−0.16**	0.04
*SISHWD*	3.30*	1.60	5.40*	2.23	0.01	0.04	0.04	0.04
*SISHWE*	3.62*	1.50	−4.18	2.23	0.17**	0.04	0.22**	0.04
*SOSHWD*	−6.04**	1.49	−14.63**	2.13	0.01	0.03	0.11**	0.03
*SOSHWE*	−2.59	1.49	14.59**	1.92	−0.04	0.02	0.03	0.03
Sense of belonging	−3.30*	1.67	−3.58	1.92	0.02	0.04	−0.09*	0.04
Teacher-student relationship	10.39**	1.70	18.39**	2.37	−0.41**	0.04	−0.25**	0.04
School leadership	1.90*	0.84	1.19	1.00	0.00	0.02	−0.05*	0.02
School size	−1.47	0.80	−2.29*	0.94	−0.01	0.02	−0.01	0.01

*p < 0.05; **p < 0.001. *ISHWD* is in-school homework time on weekdays, *ISHWE* is in-school homework time on weekends, *OSHWD* is out-of-school homework time on weekdays, and *OSHWE* is out-of-school homework time on weekends. *SISHWD*, *SISHWE*, *SOSHWD*, and *SOSHWE* are the average means of *ISHWD*, *ISHWE*, *OSHWD*, and *OSHWE* in the school level.

### 4.2. CART analysis

#### 4.2.1. Classification tree for academic scores

**Figure 1:**
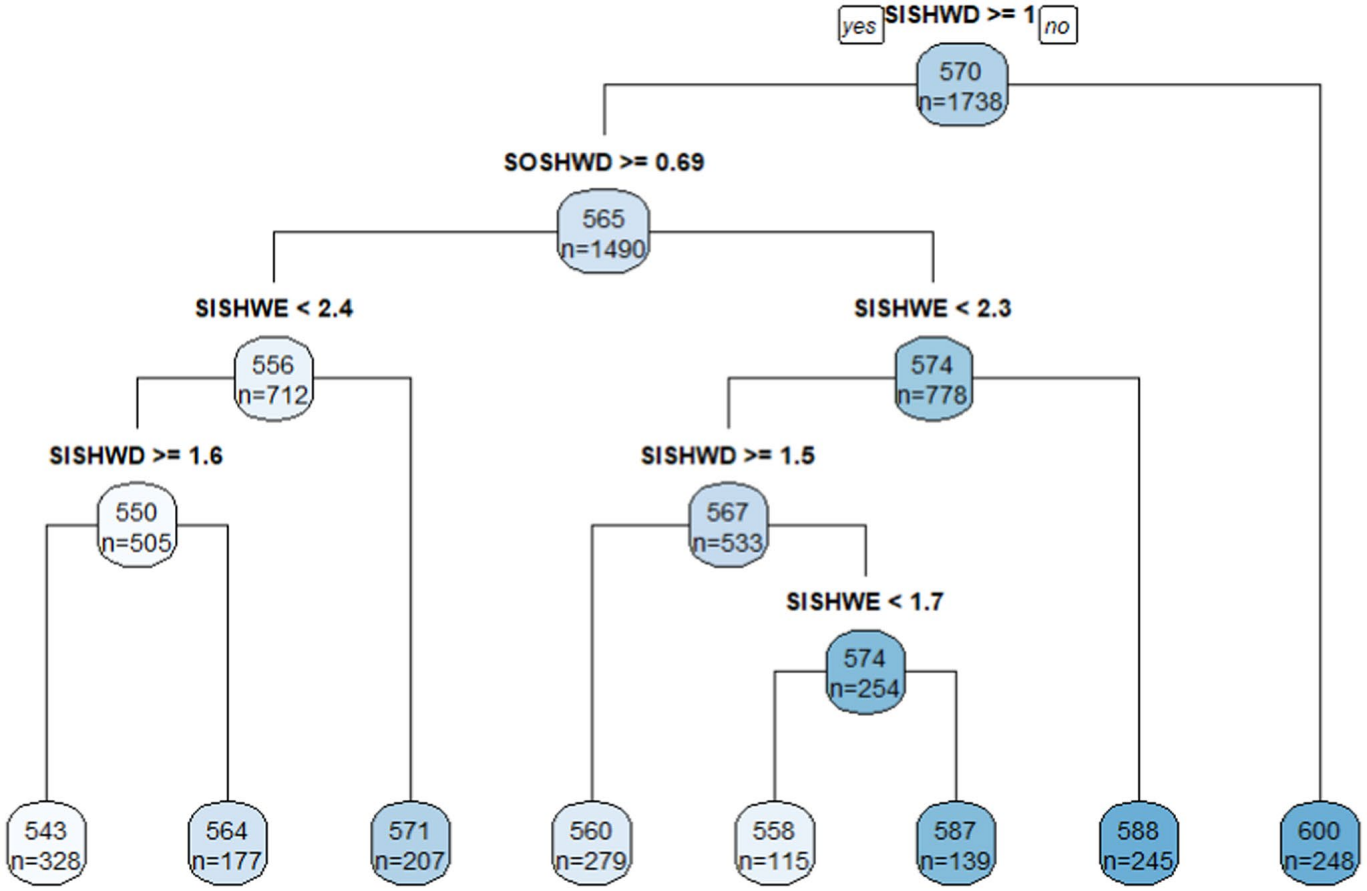
Classification tree in academic scores for Grade 4.

Using academic scores as the outcome variable, the total number of schools for Grade 4 into the CART analysis was 1,738 and the average score was 570 points. The root node was split with the most important variable *SISHWD*, the mean of which in the left node was 565 with *SISHWD* ≥ 1 h while the mean of scores in the right node was 600 with *SISHWD* < 1 h. In addition, *SISHWD* was shown in several branch nodes, both of which indicated that *SISHWD* was a primary important indicator in classifying Grade 4 students’ academic scores in the model. Moreover, these branch nodes showed that spending equal with or more than 1, 1.5 and 1.6 h on *SISHWD* were the major cutoff points that classified schools with comparable lower academic scores for Grade 4 school students. Therefore, though *SISHWD* was positively related with academic score in HLM analysis, it was showed that *SISHWD* < 1 h was associated with comparatively higher academic scores for Grade 4 students. In addition, the left child node containing 1,490 schools was split with variable *SOSHWD*. Obviously, schools with *SOSHWD* < 0.69 was linked with higher academic score (*M* = 574, *n* = 778) than those that had *SOSHWD* ≥ 0.69 h (*M* = 556, *n* = 712; Figure 1).

**Figure 2:**
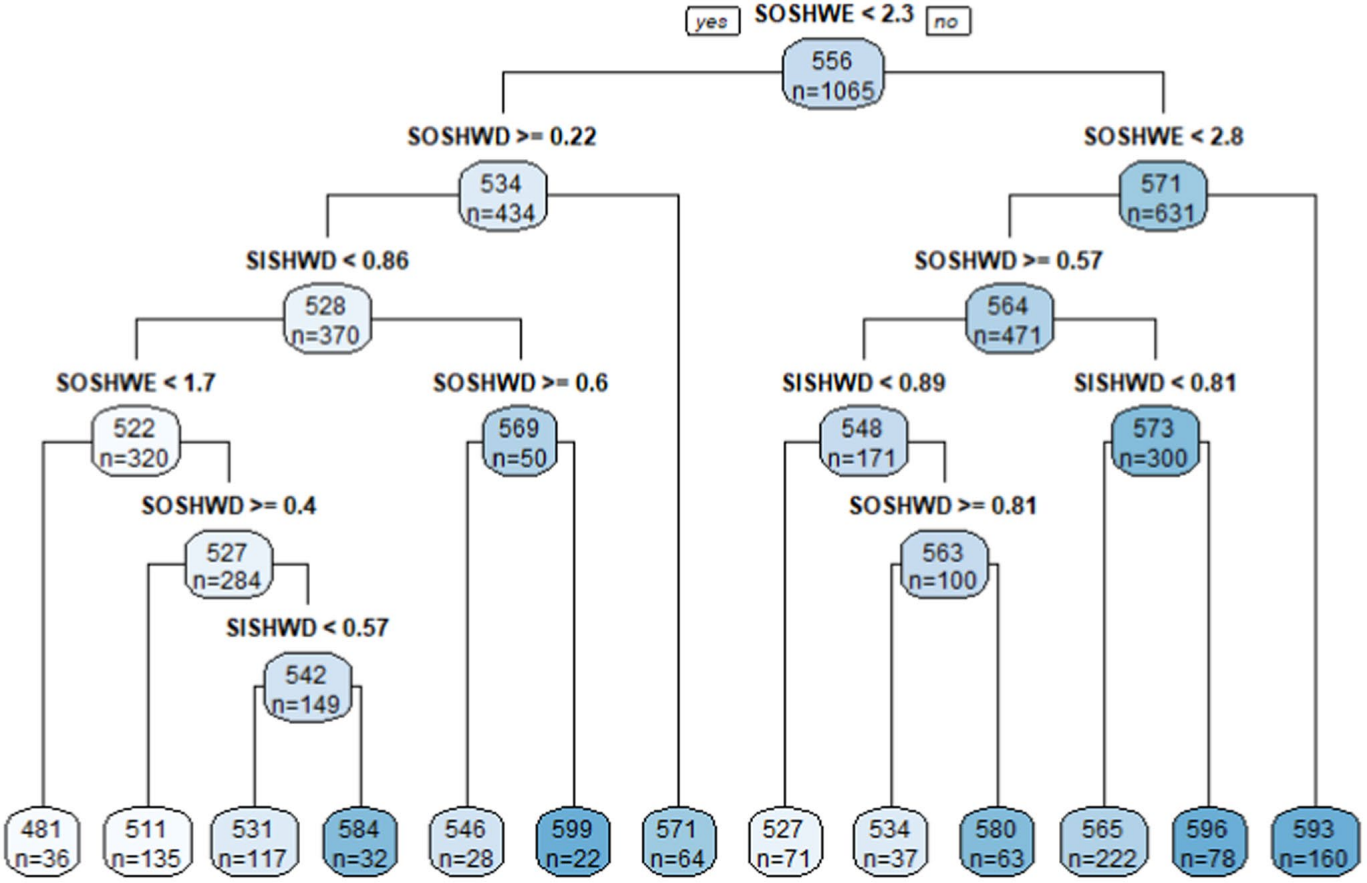
Classification tree in academic scores for Grade 8.

For the academic scores of Grade 8 schools, the tree was comparatively more complex. *SOSHWE, SOSHWD*, and *SISHWD* all showed up in the tree three to five times at multiple nodes, indicating the significant roles of these variables in dividing the students’ academic scores for Grade 8. First, *SOSHWE* was in the root node, indicating its most important role in dividing all schools into two groups. This root node resulted in a right terminal node containing 160 schools with an average academic score of 593 if *SOSHWE* ≥ 2.8 h. If, however, *SOSHWE* < 1.7 h with other conditions included, the schools’ average academic score was only 481 points. It reflected a strong positive impact of the variable *SOSHWE* on Grade 8 students’ academic scores. Second, in each branch node that had *SISHWD* as the decisive variable, the schools’ academic scores with higher *SISHWD* were higher than those with lower *SISHWD*. Since HLM also indicated that *SISHWD* was positively related with students’ academic scores for Grade 8, it validated one more time the positive relationship of *SISHWD* throughout the tree. On the contrary, in the child nodes that had *SOSHWD* as the decisive variable, the schools’ academic scores were lower with relatively high *SOSHWD* than those with low *SOSHWD*, which reflected the negative effect of *SOSHWD* on academic scores for Grade 8 (Figure 2).

#### 4.2.2. Classification tree for learning anxiety

**Figure 3:**
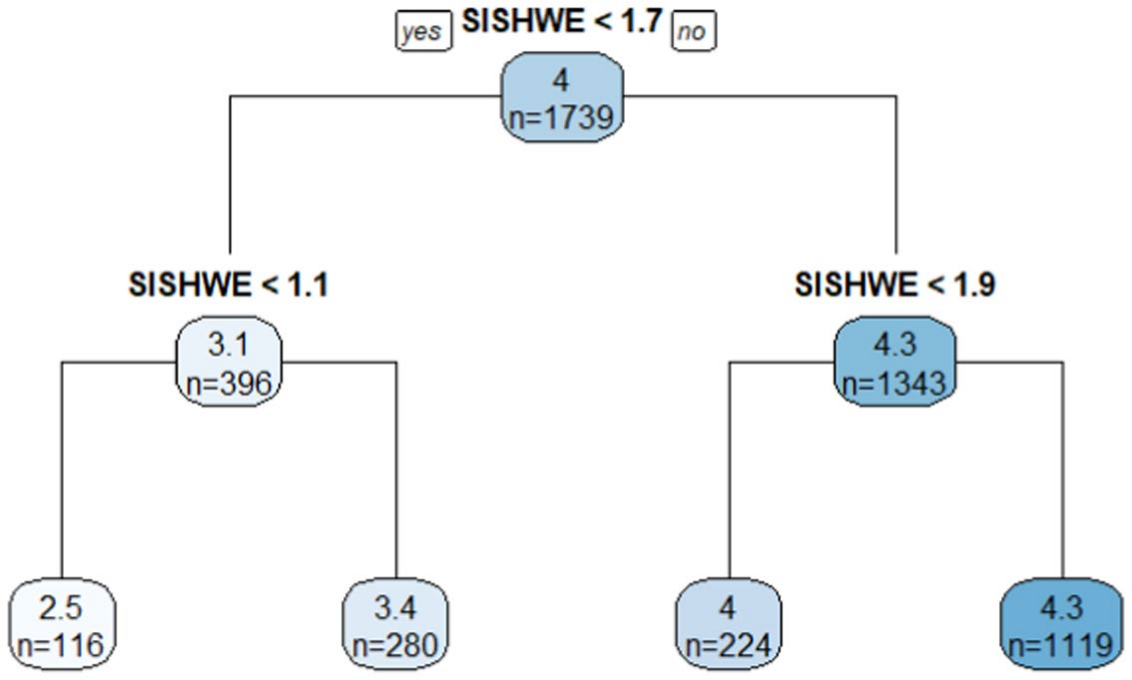
Classification tree in learning anxiety for Grade 4.

In terms of learning anxiety, the average students’ learning anxiety score for Grade 4 schools was 4. The variable *SISHWE* was the only decisive factor that related with students’ learning anxieties. When *SISHWE* ≥ 1.7 h, students’ learning anxieties in most schools in Grade 4 (*M* = 4.3, *n* = 1,343) were higher than the average (*M* = 4, *n* = 1,739), as was reflected in the right child node. In addition, there was a big contrast between the right and left terminal node. On average, the mean of school students’ learning anxieties was 4.3 when students’ time spent on in-school homework on weekends ≥1.9 (*n* = 1,119), while the mean was 2.5 when students spent <1.1 h on it (*n* = 116). However, this was only a small percentage of schools in China currently (Figure 3).

**Figure 4:**
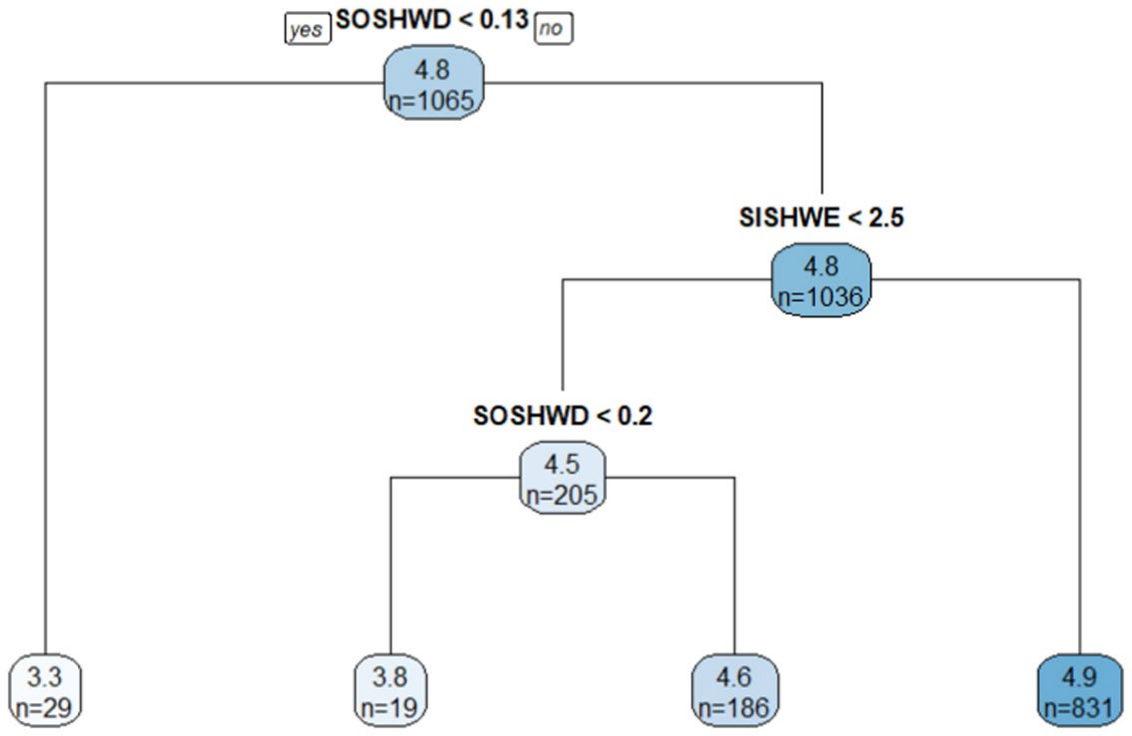
Classification tree in learning anxiety for Grade 8.

For learning anxiety in Grade 8, the classification tree was straightforward. Overall, it contained 1,065 schools, and the average score for learning anxiety was 4.8. The variable *SOSHWD* played a primary decisive role dividing Grade 8 students’ learning anxieties. There was a large contrast between the average learning anxiety in the left child but also terminal node (*M* = 3.3, *n* = 29) when *SOSHWD* < 0.13 h and the right child node (*M* = 4.8, *n* = 1,036) when *SOSHWD* ≥ 0.13 h. The right terminal node also showed that most schools (*n* = 831) had the highest learning anxieties (*M* = 4.9) when *SOSHWD* ≥ 0.13 h and *SISHWE* ≥ 2.5 h than any other interactions of variables (Figure 4).

## 5. Discussion and educational implications

This study was the first large-scale assessment that examined the effect of homework related variables on academic achievement and learning anxiety, as well as the variables’ local manifestations for Grades 4 and 8 in China. With a more nuanced classification of time spent on homework into *in-school homework on weekdays, in-school homework on weekends, out-of-school homework on weekdays* and *out-of-school homework on weekends*, it aimed to provide a more comprehensive picture and nuanced details regarding the effects of time spent on homework. Extant studies (Trautwein and Köller, 2003; Trautwein, 2007) were more of simple measures of overall time spent on homework, which cannot adequately describe the complex processes inherent in the specific homework time arrangement.

With two-level HLM and CART analyses, this study examined and confirmed the relationship of homework related variables with academic achievement and learning anxieties in the school context. There were a few significant findings identified from both analyses. First, for both Grade 4 and Grade 8, time spent on in-school homework during weekdays had positive effects on students’ academic achievement from HLM analysis. The positive effect was stronger for Grade 8 students than that for Grade 4 students, which aligns with the prior research (Muhlenbruck et al., 1999; Cooper et al., 2006; Chang et al., 2014). More detailed findings can be seen from the CART analyses as well. For Grade 4, though *SISHWD* showed a positive relationship with academic achievement, a maximum of 1 h was correlated with the highest students’ academic achievement. Therefore, except for the effects of other variables, to reach the optimal academic performance for Grade 4, more time spent on in-school homework on weekdays was recommended, but not exceeding 1 h was preferred. Comparatively, for Grade 8, one manifestation for higher academic scores from Figure 2 was more time spent on *SISHWD*, but no clear maximum time can be identified, therefore, no clear cutoff score of *SISHWD* could be recommended for Grade 8 students from the tree. In addition, since this variable had no effect on students’ learning anxiety at both grades from HLM analysis, it was one of the most efficient ways to improve students’ academic scores and there were no concerns that it would increase students’ learning anxieties. Therefore, it was appropriate to assign students certain amount of time on in-school homework on weekdays to reinforce learning content, which is beneficial for students to cultivate a self-disciplined learning habit. For Grade 4 students who had relatively lower working load in content learning, a maximum of 1 h on average was recommended; for Grade 8 students who had a much heavier load, it was recommended to assign them more amount of homework time during the weekdays. However, there are also other literature which support different opinions in the recent years (e.g., Fan et al., 2017), it is still worth exploring deeper this finding in other cultural contexts.

The second practical finding is that time spent on out-of-school homework on weekdays was negatively correlated with students’ academic achievement for both grades. The initial HLM analysis indicated the negative effect on academic achievement and its greater detrimental effect on Grade 8 than Grade 4. The follow-up CART analysis showed that for Grade 4, scores could be 574 with *SOSHWD* less than 0.69 h on average, while scores were only 556 with *SOSHWD* higher than or equal with 0.69 h. For Grade 8, students spent time on *SOSHWD* less than 0.4, 0.6, 0.57, and 0.81 h all showed a manifestation of higher scores than the groups on the other side of nodes. In addition, *SOSHWD* was statistically positively correlated with students’ learning anxieties for Grade 8 in HLM, therefore, time spent on out-of-school homework on weekdays was not recommended for either increasing students’ academic scores or lessening their learning anxieties. Combined with the results together, it was indicated that, during weekdays for either Grade 4 or Grade 8, students should develop a regulated self-disciplined habit to spend time on their in-school homework assigned by schoolteachers but not out-of-school homework. Only with appropriate arrangement and completion of in-school homework on weekdays, their academic achievement can be observed with improvement with no learning anxieties accompanied. If mixing in-school and out-of-school homework on weekdays, students will be burdened with more tasks than normal from everywhere, which brings students more pressure rather than efficiency and enjoyment from doing homework.

Whether students should spend time doing in-school or out-of-school homework on weekends is also a topic worthy our attention. In terms of time spent on in-school homework on weekends, for both grades, this factor was positively associated with student’ learning anxieties in the HLM analysis. Though it was positively correlated with Grade 4 students’ academic scores, the CART analysis (Figure 3) provided a clear cut-off score for students who had the highest learning anxieties (4.3) when time spent on in-school homework on weekends was equal with or higher than 1.9 h on average. Therefore, students should do their best to not leave their in-school homework on weekends, which is not a good option for students to increase their academic scores or lessen learning anxieties. In terms of time spent on out-of-school homework on weekends, it showed an obvious positive correlation with students’ academic scores from the HLM analysis and no impact on the learning anxieties for Grade 8. In addition, CART analysis (Figure 2) showed in the right end that *SOSHWE* higher than or equal with 2.8 h was a manifestation of the highest students’ academic achievement (*M* = 593). Several branches in the Grade 8 tree showed that higher academic scores were associated with out-of-school homework time on weekends exceeding 1.7 or 2.3 h. Moreover, since this factor did not associate with students’ learning anxieties, time spent on out-of-school homework on weekends was encouraged for Grade 8 students. On the contrary, this variable was not a factor correlated with academic scores or learning anxieties for Grade 4 students, therefore, it was not recommended for Grade 4 students to execute the same method. According to Piaget’s (1976) theory of child development stages, Grade 4 students focus mostly on image thinking and creative thinking while Grade 8 students need more time on knowledge and content learning. Therefore, it is not suitable to arrange too much out-of-school homework on weekends for Grade 4 students since on one hand, they need more extracurricular activities but not out-of-school homework to cultivate their image and creative thinking; on the other hand, Grade 4 students are not self-disciplined enough to concentrate for a specific long time, but Grade 8 students are relatively concentrated and can be assigned homework with questions focusing on deeper thinking to achieve a thoughtful and comprehensive understanding of the problems. With an increase of learning subjects and pressure to get into better high schools, the study load for Grade 8 students becomes heavier and more complex than that for Grade 4 students, therefore, it is reasonable to strengthen and consolidate the content learning through spending some time on out-of-school homework on weekends (Weiss and Kipnes, 2006). All of these results were expected to provide teachers, parents, school policymakers with more critical insights when assigning students time and types of homework.

One last thing to be noted was that, among the school-level control variables, peer relationship and teacher-student relationship both showed positive relationships with students’ academic achievement and negative association with students’ learning anxieties. It proved the significance and benefit of favorable communication with teachers and peers in students’ development academically and mentally (Ma, 2007). A harmonious interactive school environment can cultivate students with a strong sense of school belongingness and nourish them into well-rounded students.

Besides emphasizing time spent with homework (quality) and school environment, we also need to recognize the importance of quality of parent support (Dumont et al., 2014; Barger et al., 2019; Moè et al., 2020). With the quality of parent support provided, students usually would reduce much homework stress and have a better schooling experience. Moreover, though this study did not specifically target the analysis into the group of students with different disabilities (e.g., learning disability), we need to take these groups of students into consideration in the real life. Since parents of students with learning anxiety were reported more stress, need frustration than parents of typically developing students (Moè and Katz, 2018; Katz et al., 2022), the thresholds and time of range of homework for optimal outcome analyzed from this study may not apply for these groups of students with special needs. Studies with targeted groups of students might need to further understand the issue.

Overall, with a closer examination of the relationship between four homework related variables with students’ academic achievement as well as learning anxieties using a large-scale dataset in China, this study was expected to provide a deeper understanding of the potential balance between students’ academic achievement and learning anxieties. Methodologically, with the relationship identified between homework and achievement and learning anxieties and the manifestation of the variables in the classification trees, these research findings were expected to complement other statistical analyses on worldwide homework research and be of any interest to educational researchers and scholars who increasingly deal with the issue in their local settings. Practically, it was expected to provide the school executors’ data support when any early prevention or school-based intervention was executed.

## 6. Limitation

The current investigation made a valuable contribution to the incomplete body of empirical research on homework. However, still some limitations should be noted. First, though individual-level and school-level control variables were included in the study, parents’ guidance cannot be ignored. Parent facilitation was an essential mediator in the relationship between student norms, student ability, and parent attitudes toward homework (Cooper et al., 2001). Therefore, additional studies might include parent-level variables into consideration. Second, this study used students’ self-reported questionnaires as the primary data source, which carried associated risks based on variable respondent knowledge, comprehension, and interpretation of scale items. Third, homework has been measured with different scales, such as frequency (from never to every day) and effort (using three Likert-type items). With only time measured on homework, it might lead to a rather biased judgment that time is the only and most important measure of homework. More analyses with different perspectives of homework time, frequency or effect could be conducted to understand the relationships better. Also, at the student level, aside from gender and individual socioeconomic status, this study did not incorporate other important controlling variables in homework models (e.g., homework quality, feedback quality, homework expectancy, and homework effort; Cooper, 1989; Trautwein et al., 2006; Xu and Corno, 2022). These could all possibly lead to a slight change of our result. Another limitation was that we did not adopt a domain-specific approach to homework (e.g., by using average scores across multiple disciplines) since students from Grades 4 and 8 could be assigned homework besides these main disciplines, as suggested by homework models and recent studies (e.g., Trautwein et al., 2006; Xu and Corno, 2022). Without a domain-specific approach, it might not be able to provide practitioners with accurate guidance of homework time assignment in each specific discipline. Last, further research could be measured longitudinally. A large-scale study that follows a cohort of students from the early grades into adolescence would produce invaluable results. A cross-sectional study will only provide analytic results on the current state, but it is hard to infer it from a long time run.

## Data availability statement

The original contributions presented in the study are included in the article/supplementary material, further inquiries can be directed to the corresponding authors.

## Ethics statement

The studies involving human participants were reviewed and approved by all procedures performed in studies involving human participants were in accordance with the ethical standards of the institutional and/or national research committee and with the 1964 Helsinki declaration from all individual participants included in the research. Informed consent was also obtained from all individual participants included in the research. This article does not contain any studies with animals performed by any of the authors. Written informed consent to participate in this study was provided by the participants’ legal guardian/next of kin.

## Author contributions

XW and RW are in charge of the design and drafts of the manuscript. CH assists with the revision of the manuscript and provided with comments. HL and JL provided the original data, comments, and suggestions. All authors contributed to the article and approved the submitted version.

## Funding

This work was support by Yuanhui Youth Development Program: A study on the appropriateness of cognitive diagnostic assessment in Mathematics; Teacher Education “JIEBANGLINGTI” Project of Northeast Normal University: Learning Progression Construction and Learning Path Analysis Based on Cognitive Diagnosis (JSJY20220305).

## Conflict of interest

The authors declare that the research was conducted in the absence of any commercial or financial relationships that could be construed as a potential conflict of interest.

## Publisher’s note

All claims expressed in this article are solely those of the authors and do not necessarily represent those of their affiliated organizations, or those of the publisher, the editors and the reviewers. Any product that may be evaluated in this article, or claim that may be made by its manufacturer, is not guaranteed or endorsed by the publisher.
